# Announcements of Medical Lectures

**Published:** 1867-08

**Authors:** 


We are in receipt of the following announcements of Medical Lectures:—

*Berkshire Medical College*—a Summer Medical School in Pittsfield, Mass.—Commences the regular course of lectures 13th of June, and continues eighteen weeks. The lectures are conducted by *six* regular Professors, and three Adjuncts. Fees for the course, $75.00; Diploma, $20.00 Thirteen graduates last session.

*Humbolt Medical College*—St. Louis, Mo.—Has *twelve* Professors and four Adjuncts. The regular course will open on 16th Sept. next, and continue *seven* months. Fees, $105 for the lectures; $20.00 for Diploma fee; and $20.00 fee for the summer course of two months, commencing 15th May. Four graduates at last session.

*Medical College of Virginia*—Richmond, Va.—Has 8 Professors—Opens regular course 1st October, and continues five months. A summer preparatory course is held in the college, commencing 1st April, and ends the last of July. Fees for the regular winter course, $120; Diploma fee, $30.00. Sixty dollars is charged for the summer course, one half of which will be deducted from fees of the succeeding winter course. Twenty applicants had the degree conferred at the last session.

*Rush Medical College*—Chicago, Ill.—Has 8 Professors—Commences its session 3d Oct., and continues eighteen weeks. A summer preparatory course commences 1st Wednesday of March. Fees for the regular course of lectures, $50.00; Graduation, $25.00. At the last session seventy-one graduated in this institution.

*Medical College of the State of South Carolina*—Charleston, S. C.—Opens its next annual session the 1st of Nov., and continues four months. The Faculty consists of *seven* regular and *six* Adjunct Professors. Fees for the course, $105; Graduation, $30.00. Thirty-one received the degree at the end of the last session.

*Medical Department of the University of Louisiana*—New Orleans, La.—Has 7 Professors—Commences its course about the 1st of Nov., and continues four months. Fees for the course of lectures, $140; Graduation, $30.00. Seventy-two graduates for last session.

*Washington University, Medical Department*—Baltimore, Md.—Has 9 Professors—Opens the first course of lectures about the 1st of Oct. next, and continues five months. Fees for the course of lectures, $120; Graduation, $20.00. A summer course, supplementary to the regular winter course, commencing first Monday of April, and concluding the last of July, will be conducted by an Adjunct Faculty of seven Professors.

